# Supplementary material for: Lygistorrhinidae (Diptera: Bibionomorpha: Sciaroidea) in early Eocene Cambay amber
Source: PeerJ. 2017 May 17;5:e3313. doi: 10.7717/peerj.3313 (PMC5437858; doi:10.7717/peerj.3313)
Supplement: Supplemental Information 1 [file peerj-05-3313-s001.pdf]

Supplemental Material

Table S1. Data matrix for phylogenetic analysis of Lygistorrhinidae.

	1	2	3	4	5	6	7	8	9	10	11	12	13	14	15	16	17	18	19	20	21	22	23	24	25	26	27	28	29	30	31	32	33	34	35	36	37	38	39	40	41	42	43	44	45	46	47	48	49	50	51	52	53	54	55	56	57	58	59	60
01	0	2	0	?	0	0	0	0	0	0	0	0	1	0	0	1	0	2	0	1	0	0	0	0	1	0	0	0	0	0	0	0	0	0	0	0	0	0	0	0	0	0	1	0	0	0	0	1	0	0	0	0	0	0	0	0	0	2	0	1
02	0	2	0	0	0	0	?	0	1	0	0	0	0	0	0	0	0	0	0	0	-	0	1	0	0	0	0	0	0	0	0	0	1	0	0	0	0	0	0	0	0	0	?	0	0	0	0	1	1	0	?	?	0	?	1	0	0	-	?	0
03	0	2	1	0	0	0	?	?	?	0	0	0	0	0	0	0	?	?	0	0	-	0	2	1	0	0	1	0	0	0	0	0	0	0	0	0	0	0	0	?	1	1	?	0	0	0	0	?	0	?	0	0	0	?	1	1	0	0	?	1
04	0	2	2	0	0	0	0	0	1	0	0	0	0	0	0	0	0	1	0	0	-	0	1	0	0	1	1	0	1	1	0	0	0	0	0	0	0	0	1	1	1	1	?	0	0	0	1	?	0	0	0	0	0	?	1	1	1	-	?	0
05	0	2	1	0	0	0	0	0	1	0	0	0	0	0	0	0	0	1	0	1	0	0	1	1	0	1	1	0	1	0	0	0	1	0	0	0	0	0	1	1	1	1	?	0	0	0	1	1	?	0	?	?	0	?	0	1	1	-	?	0
06	0	2	0	0	0	0	0	0	0	0	0	0	0	0	0	0	0	1	0	1	1	0	0	0	0	0	1	0	1	0	?	?	1	0	0	0	0	0	1	1	0	0	?	0	0	0	0	1	?	0	0	0	0	?	1	0	0	1	?	0
07	0	0	3	0	0	0	0	1	0	0	0	0	0	0	0	0	0	1	0	0	-	?	0	0	1	0	1	0	0	1	0	0	1	0	0	0	0	0	1	1	0	1	?	0	0	0	1	1	?	1	1	1	1	?	1	0	0	2	0	1
08	0	0	3	0	0	0	0	1	0	0	0	0	0	0	0	0	0	1	0	0	-	0	0	0	1	0	0	0	0	1	0	0	1	0	0	0	0	0	1	1	0	1	?	0	0	0	1	0	?	1	1	1	1	?	1	0	0	?	?	1
09	1	0	3	1	0	0	0	1	0	0	0	0	0	1	1	0	0	2	0	1	0	0	0	0	2	0	1	1	-	1	0	1	1	0	0	0	0	0	0	0	0	0	2	1	0	0	0	0	?	0	0	0	0	2	1	0	0	2	1	1
10	1	0	3	1	0	0	0	1	0	0	1	0	0	1	1	0	0	2	0	1	0	0	0	0	2	0	1	1	-	1	0	1	1	0	0	0	0	0	0	1	0	0	2	1	0	0	?	?	?	?	0	0	0	2	1	0	0	2	1	1
11	2	1	3	0	1	0	0	1	1	0	0	0	0	1	1	1	1	1	1	1	1	0	1	0	2	0	1	1	-	1	0	0	1	0	0	0	0	1	1	1	0	0	?	0	0	1	1	0	0	0	0	1	0	1	1	0	0	1	3	1
12	2	1	3	0	0	0	0	1	1	0	0	0	1	1	1	1	1	1	1	1	1	0	1	1	2	0	1	1	-	1	?	?	?	1	0	1	1	1	1	1	1	1	?	0	0	0	1	0	?	0	?	?	1	1	1	0	0	1	3	1
13	1	0	3	0	0	1	0	1	0	0	0	1	0	0	0	1	0	1	1	1	0	0	1	1	2	0	1	1	1	1	0	1	1	1	1	0	0	0	1	1	0	1	0	0	0	0	1	1	0	0	1	1	1	1	1	1	0	1	2	0
14	1	0	3	0	0	1	1	-	-	1	1	1	0	0	0	1	0	1	1	1	1	0	1	1	2	0	1	1	-	1	0	0	1	1	2	0	0	0	1	1	?	0	0	0	1	1	1	0	1	0	1	0	0	1	1	1	0	1	2	1
15	0	0	3	1	0	0	1	-	-	0	2	1	1	0	0	0	0	1	1	[01]	0	0	1	1	2	0	1	0	1	1	1	1	1	0	1	0	0	0	1	1	?	0	0	0	1	1	1	0	1	0	0	0	0	1	1	1	0	1	2	1
16	1	0	3	0	1	0	1	-	-	2	0	1	1	1	1	0	0	2	0	0	-	1	1	1	2	0	1	1	-	1	1	1	1	1	2	0	0	0	1	1	0	0	0	0	1	1	1	0	?	1	?	?	1	1	1	1	0	0	?	1
17	2	0	3	0	1	0	1	-	-	0	0	1	1	1	1	1	0	1	0	0	-	0	1	0	2	0	1	1	-	1	0	0	1	0	0	0	0	0	1	1	1	0	0	0	1	1	1	0	0	1	1	1	0	1	1	1	0	1	2	1
18	0	0	3	0	1	1	1	-	-	2	0	1	0	1	1	1	0	1	0	0	-	1	1	0	2	0	0	0	1	1	1	1	1	1	2	1	1	0	0	?	1	0	0	0	0	0	1	0	0	1	?	?	0	?	1	1	0	1	0	1
19	0	2	0	1	1	1	0	1	1	0	0	0	0	0	0	0	0	1	0	0	-	0	0	0	1	0	1	0	0	0	0	1	1	0	0	0	0	0	1	1	2	1	0	1	0	0	0	0	0	0	0	0	-	?	1	-	-	-	-	1
20	2	0	3	?	-	0	0	1	1	0	0	0	0	0	0	0	0	0	1	1	0	0	1	0	1	0	0	1	-	1	?	?	1	0	0	0	0	0	1	0	0	1	0	-	0	0	0	1	0	0	0	0	-	-	-	-	-	-	-	1

01 - *Diadocidia ferruginosa*
02 - *Archaeognoriste*
03 - *Lebanognoriste*
04 - *Plesiognoriste zherikhini*
05 - *Protognoriste amplicauda*
06 - *Leptognoriste davisi*
07 - *Palaeognoriste sciariforme*
08 - *Palaeognoriste affine*
09 - *Asiorrhina asiatica*
10 - *Asiorrhina parasiatica*
11 - *Lygistorrhina sanctaecatherinae*
12 - *Lygistorrhina fijiensis*
13 - *Labellorrhina quantula*
14 - *Blagorrhina blagoderovi*
15 - *Gracillorrhina gracilis*
16 - *Loyugesa khuati*
17 - *Matileola yangi*
18 - *Seguyola sp.*
19 - *Parisognoriste eocenica*
20 - *Indorrhina sahnii*
